# Randomized clinical trial comparing percutaneous closure of patent foramen ovale (PFO) using the Amplatzer PFO Occluder with medical treatment in patients with cryptogenic embolism (PC-Trial): *rationale and design*

**DOI:** 10.1186/1745-6215-12-56

**Published:** 2011-02-28

**Authors:** Ahmed A Khattab, Stephan Windecker, Peter Jüni, David Hildick-Smith, Dariusz Dudek, Henning R Andersen, Reda Ibrahim, Gerhard Schuler, Antony S Walton, Andreas Wahl, Heinrich P Mattle, Bernhard Meier

**Affiliations:** 1 Cardiology Department, Bern University Hospital, Bern, Switzerland; 2 Neurology Department, Bern University Hospital, Bern, Switzerland; 3 Institute for Social Medicine, Bern University, Bern, Switzerland; 4 Sussex Cardiac Center, Sussex and Brighton University Hospital, Brighton, UK; 5 Department of Interventional Cardiology, University College, Krakow, Poland; 6 Department of Cardiology, Aarhus University Hospital, Aarhus, Denmark; 7 Department of Cardiology, Montreal Heart Institute, Montreal, Canada; 8 Heart Center, University of Leipzig, Leipzig, Germany; 9 Department of Cardiology, Alfred Hospital, Prahran, Australia

## Abstract

**Background:**

Several studies have shown an association of cryptogenic stroke and embolism with patent foramen ovale (PFO), but the question how to prevent further events in such patients is unresolved. Options include antithrombotic treatment with warfarin or antiplatelet agents or surgical or endovascular closure of the PFO. The PC-Trial was set up to compare endovascular closure and best medical treatment for prevention of recurrent events.

**Methods:**

The PC-Trial is a randomized clinical trial comparing the efficacy of percutaneous closure of the PFO using the Amplatzer PFO occluder with best medical treatment in patients with cryptogenic embolism, i.e. mostly cryptogenic stroke. Warfarin for 6 months followed by antiplatelet agents is recommended as medical treatment. Randomization is stratified according to patients age (<45 versus ≥45 years), presence of atrial septal aneurysm (ASA yes or no) and number of embolic events before randomization (one versus more than one event). Primary endpoints are death, nonfatal stroke and peripheral embolism.

**Discussion:**

patients were randomized in 29 centers of Europe, Canada, and Australia. Randomization started February 2000. Enrollment of 414 patients was completed in February 2009. All patients will be followed-up longitudinally. Follow-up is maintained until the last enrolled patient is beyond 2.5 years of follow-up (expected in 2011).

**Trial Registration:**

Trial listed in ClinicalTrials.gov as NCT00166257 and sponsored by AGA Medical, Plymouth, MN, USA

## Introduction

The cause of ischemic stroke is considered cryptogenic in 35-40% of cases [1]. Paradoxical embolism can be the cause of systemic embolism under all circumstances provided there is a right to left shunt, but it is blamed for an embolic event typically only in the absence of a left-sided thromboembolic source. Hence, paradoxical embolism via a patent foramen ovale (PFO) is entertained in the differential diagnosis especially in young patients (<60 years old) [2]. The detection of thrombus in the venous system or right atrium is not to be considered a prerequisite as non-detectable small clots are the most common culprits. Since the direct detection of thrombus within a PFO is rare [3-6], the diagnosis of paradoxical embolism is usually presumptive. While there is currently no proof for a cause-effect relationship, several studies have confirmed a strong association between the presence of a PFO and the risk for paradoxical embolism or stroke [7-11]. A PFO with a coinciding atrial septal aneurysm (ASA), spontaneous or large right-to-left shunt, or multiple ischemic events potentiates the risk of recurrence. [12]. The presence of a PFO or ASA has currently no therapeutic consequence in otherwise healthy adults. In contrast, patients suffering a stroke or transient ischemic attack (TIA) in the presence of a PFO are to be considered for medical therapy to reduce the risk of a recurrent embolic event (secondary prevention). Patients with PFO and ASA are defined as 'high-risk' patients for recurrent events. A retrospective French multicenter study in such patients reported a yearly risk of 1.2% to sustain a recurrent stroke and of 3.4% to suffer a recurrent stroke or TIA despite medical treatment with antiplatelet drugs or oral anticoagulants in patients with PFO and cryptogenic stroke [13]. In the Lausanne study no differences in risk reduction were noted between different modes of anticoagulant therapy with respect to recurrent stroke and TIA [14]. The Quality Standards Subcommittee of the American Academy of Neurology recently published evidence-based guidelines for the management of patients with recurrent stroke, PFO, and ASA, after a critical review of the literature [15]. Their main conclusion was that PFO alone does not portend an increased risk of subsequent stroke or death in patients who have had a cryptogenic stroke and are treated medically [15]. There were insufficient data to draw conclusions about isolated ASA. The results regarding patients with the combination of PFO and ASA were somewhat inconsistent and did not allow drawing conclusions for this subset of patients.

Nonsurgical closure of PFO has become possible with the advent of implantable devices, and has long been demonstrated feasible and safe [16,17]. It represents an attractive treatment alternative to life-long antiplatelet therapy or oral anticoagulation in patients with PFO and paradoxical embolism.

The optimal treatment strategy of such symptomatic patients remains to be defined, although nonrandomized data suggest an advantage of percutaneous PFO closure over medical treatment [18,19]. Thus the true therapeutic efficacy of percutaneous PFO closure as an adjunct or alternative to medical treatment needs to be ascertained by randomized studies.

## Methods

### Rationale for the present study

While there is good evidence for the association between the presence of PFO and the risk for paradoxical embolism, a cause-effect relationship has not been conclusively established. Furthermore there are no publications of prospective, randomized studies comparing the efficacy of different treatment modalities in patients with PFO and paradoxical embolism including percutaneous device closure.

The purpose of the present study is to compare the efficacy of percutaneous PFO closure using the *Amplatzer PFO Occluder *(AGA Medical Corporation, Plymouth, MN, USA), with medical treatment in patients with presumed paradoxical embolism in a prospective, randomized trial.

### Null-hypothesis to be rejected

Percutaneous device closure of PFO with the *Amplatzer PFO Occluder *is of equal efficacy compared with medical treatment in the prevention of recurrent stroke and/or TIA in patients with paradoxical embolism.

### Design overview

This is a multicenter, multinational, randomized, clinical trial comparing the efficacy of percutaneous PFO closure using the *Amplatzer PFO Occluder *with best medical treatment in patients with PFO and paradoxical embolism. The organization and scientific conduct of the study is supervised by a Steering Committee. A Data and Safety Monitoring Board is responsible for safety and ethical aspects. A Clinical Events Committee review and adjudicate all reported adverse events and endpoints. Study monitoring and data management is carried out by a contract research organization.

### Study endpoints

The primary objective of this study is to investigate whether percutaneous PFO closure using the *Amplatzer PFO Occluder *is superior compared to best medical treatment in the prevention of symptomatic, recurrent thromboembolism. The following combined primary endpoint will therefore be assessed at each scheduled follow-up visit: death, nonfatal stroke, and peripheral embolism.

Patients who experience a primary endpoint event during the study period no longer have to continue the treatment allocated at the time of randomization. The management of patients who suffer a primary endpoint event will be left to the discretion of the responsible physician. Crossover from medical treatment to PFO device closure or vice versa from PFO device closure to additional antithrombotic treatment will be allowed in case of a primary endpoint event. However, all patients will continue to be followed up for further events after an initial primary endpoint according to the study protocol for the total duration of this study. Appendix 1 details the primary and secondary endpoints.

### Patient selection criteria

Patients with PFO and presumed paradoxical embolism who meet all inclusion criteria but no exclusion criteria of Appendices 2 and 3 are offered to participate in this study. After obtaining a written informed consent, patients were randomised in 29 centers of Europe, Canada, and Australia. Randomization started February 2000. Enrolment of 414 patients was completed in February 2009. All patients will be followed-up longitudinally as outlined in Table 1. Follow-up is maintained until the last enrolled patient is beyond 2.5 years of follow-up (expected in 2011).

**Table 1:** Timetable of prospective investigations during the study.

	Baseline	Hospitalization	6 months	1 year	2 years	3 years	4 years	5 years
History	x		x	x	x	x	x	x
Listing of antithrombotic medications	x	x	x	x	x	x	x	x
Laboratory survey	x							
12 lead ECG	x	x	x	x	x	x	x	x
Holter ECG	x							
TEE*	x	X (TTE or TEE)	x					(x)
CT/MR or angiography of region of interest (brain or periphery)	x							
Carotid Doppler	x							
Employment status	x		x	x	x	x	x	x
Quality of life	x		x	x	x	x	x	x
Stroke outcome classification (NIH SS, Barthel index, Modified Rankin scale)	x	x	x	x	x	x	x	x
Procedural complications		x						
Primary endpoints			x	x	x	x	x	x
Secondary endpoints			x	x	x	x	x	x

*TEE examination after 6 months and possibly yearly thereafter until complete closure of the PFO has been documented. A TEE at 5 years of follow-up or termination of the study is optimal; a TTE is acceptable immediately following device closure.

### Treatment assignment

Randomization was done by a research contract organization (InterCorNet, Zurich, Switzerland) through computer-generated block randomisation forms for each participating centre. Patients were stratified according to age (≥45 vs. <45 years) and presence or absence of ASA. Percutaneous PFO closure was scheduled as soon as possible following randomization. Implantation occurred according to standard techniques by experienced operators familiar with the Amplatzer PFO Occluder.

### Device

The device used for percutaneous PFO closure in patients participating in the *PC-*trial was the *Amplatzer PFO Occluder*, manufactured from 0.005" nitinol wire, a superelastic shape memory alloy, which permits constraining of the device to a small (≤9 French) delivery system with recovery of shape upon delivery in the atria. As long as the device is screwed to its 0.038" delivery cable, it is fully retrievable and repositionable without need for removal of the device from the delivery sheath prior to a new placement attempt. The device is available in different sizes with typically the left atrial disk being smaller than the right atrial disk (18 mm to 35 mm).

### Implantation procedure

Patients scheduled for percutaneous PFO closure were generally admitted on the day of the procedure and discharged the same or following day. The procedure was generally performed using local anesthesia. Balloon gauging of the PFO was not encouraged. The decision to guide device implantation by fluoroscopy only or use simultaneous transesophegeal echocardiography (TEE) or intracardiac echocardiography (ICE) was left to the implanting physician. Prophylactic antibiotic therapy (e.g. cephalosporin) was recommended during the periprocedural period followed by a recommendation for prophylaxis against endocarditis for 2-6 months. Venous access was typically established via the right femoral vein. Femoral artery access was optional for invasive blood pressure monitoring or to assess for coronary artery disease by diagnostic coronary angiography. In case of left-to-right shunt (small atrial septal defects) balloon gauging and a complete right heart catheterization with oximetry were recommended but not mandatory. After administration of systemic heparin (≥ 5000 IU) the PFO is probed, e.g. with a multipurpose catheter or a standard (exchange) guidewire (0.035 inch). A 8-9 French Amplatzer sheath was used for device implantation. The *Amplatzer PFO Occluder *was delivered through the delivery sheath and placed within the PFO under fluoroscopic and optional echocardiographic guidance. First, the usually smaller left atrial disc was released in the left atrium. The whole unit consisting of the sheath and the released left atrial disc was then pulled gently against the interatrial septum. Following this the right atrial disc was released by further withdrawing the sheath, while maintaining some traction on the interatrial septum with the left atrial disc. Care was taken to avoid air embolism. After assurance of a stable and correct device position (fluoroscopy with dye injection in a view showing the device in profile separating the 2 disks completely or echocardiography), the device was released from the delivery cable. The final position and eventual residual shunt were documented by angiography or echocardiography. Following this, the introducer sheath was removed and hemostasis assured by applying manual compression to the access site. Before hospital discharge stable device position was documented by transthoracic echocardiography (TTE). All implantation related complications were recorded as adverse events.

A residual shunt is not unexpected immediately following device implantation. Subsequent endothelialization occurring over a few months improves the closure rate. Completeness of closure for analysis was therefore assessed by TEE with Valsalva followed by a bubble test at 6 months of follow-up. All patients to be treated with acetylsalicylic acid 100-325 mg/day for 5-6 months following device implantation. Ticlopidine 250-500 mg/day or clopidogrel 75-150 mg/day, could be used complementary or as an alternative in case of intolerance to acetylsalicylic acid. Oral anticoagulation was optional for a period of 6 months, if deemed necessary by the investigator but was discouraged. Provided a satisfactory result of the 6-month contrast TEE, antithrombotic therapy and the recommendation for prophylaxis against endocarditis were discontinued, unless required by another indication.

### Best medical treatment

Patients randomized to the best medical treatment arm were not undergoing any catheterization procedure but were treated with antithrombotic medication. Since there is no consensus on the most effective medical therapy in preventing recurrent stroke or TIA in patients with PFO and paradoxical embolism, the choice of antithrombotic therapy, either antiplatelet therapy or oral anticoagulation, was left to the discretion of the treating physician. However, all patients randomized to the best medical treatment arm had to be treated with at least one antithrombotic medication. The recommended antithrombotic regimen in this study was oral anticoagulation for a period of 6 months (to be commenced at the time of inclusion in the study), followed by treatment with acetylsalicylic acid 100 mg to 325 mg per day during the remainder of the study. However, alternative treatment regimens using only oral anticoagulation or antiplatelet drugs or a combination of these medications were specifically allowed as considered necessary by the responsible physician. This is in keeping with the goal of the study to compare percutaneous PFO closure against best medical treatment, meaning medical treatment deemed optimal for the individual patient by the responsible physician. The recommended therapeutic range for patients who are orally anticoagulated in this study was an international normalized ratio (INR) of 2.0-3.0. A crossover from oral anticoagulation to antiplatelet therapy with acetylsalicylic acid, ticlopidine, or clopidogrel or vice versa was allowed within the best medical treatment arm. The date of drug therapy change and the reasons was documented in the case record form (CRF). Furthermore, events leading to interruption of antithrombotic therapy or bleeding complications were recorded as adverse events in the follow-up section of the CRF. Medical treatment was commenced immediately after randomization, unless already en route. Any events leading to interruption of antithrombotic therapy as well as any bleeding complications were recorded as adverse events.

The statistical power of this study was based on the population allocated to the best medical treatment group as a whole. Although differences among the different antithrombotic regimens will be analyzed, the statistical power may be inadequate to allow firm respective conclusions.

### Adverse events

#### Risk of percutaneous PFO closure

The risks of percutaneous PFO closure using the *Amplatzer PFO Occluder *consist of the risk of the implantation procedure and the long-term risk of the device itself. The implantation risks are similar to those of other interventional cardiac procedures [20]. They include complications related to vascular access (1-2%; e.g. bleeding, hematoma, need for transfusion), cardiac perforation with or without tamponade (<1%) [21-26], or air embolism (1-2%). The risks of stroke and myocardial infarction are expected to be lower than with coronary angiography which requires arterial access. The risk of death is minimal.

Complications intrinsic to the PFO device include device embolization (3-6%) primarily into the right circulation. Usually, the device can be retrieved either percutaneously, rarely surgery is needed. Other potential hazards include thrombus formation in the implantation equipment or on the device surface with the risk of subsequent embolization, infective endocarditis, or device collapse due to structural fatigue. Bleeding due to the post implantation medical management is another hazard.

#### Risk of medical treatment

The risks of medical treatment relate to adverse events of antithrombotic therapy. Bleeding is the main complication of oral anticoagulant therapy with warfarin or alike [27,28] and is influenced by the intensity of anticoagulation and the concomitant use of antiplatelet agents. The bleeding risk appears higher in patients with a history of prior stroke or gastrointestinal bleeding. Intracranial hemorrhage is estimated to occur with an incidence of 0.5% per year in patients on oral anticoagulant therapy with coumadin. Another important, but uncommon adverse effect of coumadin is skin necrosis. Furthermore many other medications and food components interfere with the metabolism of oral anticoagulants, potentiating or inhibiting their effects.

The principal adverse effects of acetylsalicylic acid are prolonged bleeding due to inhibition of platelet function, stomach irritation, gastritis, or gastrointestinal bleeding. These complications are rare and the therapeutic benefit of acetylsalicylic acid outweighs these potential adverse advents. Acetylsalicylic acid increases the risk for intracranial hemorrhage minimally. In case of intolerance to acetylsalicylic acid, ticlopidine or clopidogrel were used. Adverse event definitions for both treatment modalities are shown in Appendix 4.

### Statistical Methods

The working hypothesis of this study postulates a reduction in the annual incidence of recurrent thromboembolic events from 3% per year to ≤1% per year in patients with percutaneous closure of the PFO. With an overall sample size of 410, the power will be 80% at an α-level of 0.0492 (allowing for one interim analysis) under the following assumptions: mean follow-up time 4.5 years; successful closure of the PFO in 95% of those randomized to PFO closure; and annual rate of patients lost to follow-up 0.5%. The comparability between the two treatment groups will be tested with the two sample t-test or Fischer's exact test as appropriate, for continuous covariates, and Pearson's chi square test for categorical variables. An adjusted alpha level of 0.01 will be used as indication for an imbalance between the study groups.

The data from all participating centers will be pooled for the analysis of all primary and secondary endpoints as well as adverse events. An intention-to-treat analysis will be carried out retaining all the randomized study participants irrespective of whether they received the allocated treatment or not. Analysis of primary and secondary end points will be done using proportional hazard models. Graphical representation and comparison of survival between the two intervention groups will be presented using Kaplan Meier survival curves. Secondary analyses of these end points stratified by age, (age ≥45 years vs. <45 years and presence or absence of ASA) and effect modification by age and ASA will be tested in multivariable modeling. The significance level to reject the null hypothesis is set to 0.0492 (two-sided) taking account of the planned interim analysis.

An interim analysis will be conducted after 2.5 years of follow-up of the entire study population to detect an important excess in benefit or risk of closure of the PFO as compared to medical treatment. The significance level to define statistically a significant excess or reduction in risk will be 0.0054 (two-sided) according to O'Brian-Fleming boundaries. Data and Safety Monitoring Board is entitled to interrupt the trial taking into account both, clinical and statistical significance of excess or reduction in risk.

### Study organization

The study comprises the participating centres and an organizing board structure that includes a Steering Committee, a Data and Safety Monitoring Board, a Data Coordinating Center, and a Statistical Core Center. Each participating centre has a nominated research assistant to supervise the study and organize local data collection. This study is conducted in compliance with the Helsinki Declaration and an ethical approval was given from the responsible ethical committee of every participating centre.

## Discussion

In most people, a PFO will remain asymptomatic for life. However, since the initial description of a fatal stroke in a young woman linked to a PFO by Cohnheim in 1877, PFO and ASA have been increasingly recognized as potential mediators of several disease manifestations, including paradoxical embolism with the principal risk of cerebral or myocardial infarction, orthostatic desaturation in the setting of the rare platypnoea-orthodeoxia syndrome, refractory hypoxemia due to right-to-left shunt in patients with right ventricular infarction or severe pulmonary disease, neurological decompression illness in divers, migraine with aura, transient global amnesia, obstructive sleep apnea, and high-altitude pulmonary edema.

Several case-control studies using contrast echocardiography showed a strong relation between the presence of PFO and cryptogenic stroke in adults particularly aged <55 years [29-34]. According to a meta-analysis [35], in patients younger than 55 years, a PFO confers a relative risk of 3 (95% CI 2 to 4) comparing ischemic stroke with non-stroke control subjects, and a relative risk of 6 (95% CI 4 to 10) comparing cryptogenic stroke with control subjects with a known cause of stroke.

Medical treatment using antithrombotic drugs, percutaneous device closure, or surgical closure constitute measures for secondary prevention against recurrent embolic events in patients with PFO. To date, published randomized comparisons between these modalities regarding safety and efficacy are lacking.

At present, the most restrictive indications are applied in the USA, where failed medical treatment for secondary stroke prevention constitutes the sole Food and Drug Agency accepted indication for PFO closure with a plea to effect the treatment exclusively in the realm of a randomized study [36].

## Appendix 1: PC-trial endpoints

### Primary composite endpoint

a. Death

1. Fatal stroke: a stroke deemed to have caused death either directly by brain damage or indirectly by some non-neurological complication.

2. Cardiovascular death: including sudden death, myocardial infarction, circulatory failure, pulmonary edema, heart failure and death due to any vascular cause including aortic, mesenteric, or peripheral vascular or embolic disease. An unwitnessed unsuspected death will be counted as sudden death.

3. Non-cardiovascular death: any death not classified as fatal stroke or cardiovascular death.

**b. Nonfatal stroke**: any neurologic deficit lasting for >24 hours.

1. Major stroke: a new neurologic deficit that persists for more than 7 days and increases the NIH Stroke Scale score by >4.

2. Minor stroke: a new neurologic deficit that either resolves completely within 7 days or increased the NIH Stroke Scale score by less than <3.

3. TIA: a transient ischemic attack is defined as a temporary neurologic deficit presumably due to reduced blood flow in a particular cerebral artery lasting for <24 hours with complete resolution of the neurologic deficit.

**c. Peripheral embolism**: any endorgan ischemia other than in the brain caused by reduced blood flow in a particular artery and objectively documented by Duplex, computed tomography (CT), MR imaging, or angiography.

### Secondary endpoints

a. New arrhythmia: any rhythm disorder other than normal sinus rhythm, requiring either hospitalization or pharmacologic or electrical therapy interventions.

b. Myocardial infarction: CK-MB >3 times the upper limit of normal and present in two separate blood samples or new pathological Q-waves in at least two contiguous leads with any elevated CK-MB.

c. Rehospitalization related to PFO or its treatment: >24 hour in-hospital stay, which becomes necessary for the management of complications related either to the PFO or antithrombotic therapy.

d. Device problems (dislodgment, structural failure, infection, thrombosis etc.)

e. Bleeding complications related to antithrombotic therapy will be classified as severe, if requiring any blood transfusion, and minor, if not requiring blood transfusions.

## Appendix 2: PC-trial inclusion criteria

a. Age <60 years old

b. Presence of PFO (with or without ASA) documented by TEE with a right-to-left shunt during the bubble test or color Doppler flow imaging either spontaneously or with a Valsalva or cough maneuver.

c. Ischemic stroke verified clinically and neuroradiologically by magnetic resonance, computed tomography or angiography in the absence of another identifiable cause of stroke (see exclusion criteria).

d. Symptoms of TIA and neuroradiologically identified intracranial ischemic lesion in the absence of another identifiable cause of stroke (see exclusion criteria).

e. Clinically and radiologically verified extracranial peripheral thromboembolism in the absence of another identifiable cause of thromboembolism (see exclusion criteria).

f. Sufficient recovery from the thromboembolic index event to allow independent daily activities.

g. The physician implanting the device in case of allocation to percutaneous PFO closure agrees to implant an Amplatzer PFO Occluder Other commercially available devices for percutaneous PFO closure are not to be used in this study.

## Appendix 3: PC-trial exclusion criteria

a. Any identifiable cause for the thromboembolic event other than PFO. The following causes must be specifically excluded in all patients enrolled in this study:

***Cardiac: ***mural thrombus, dilated cardiomyopathy, prosthetic heart valve, mitral stenosis, bacterial and nonbacterial endocarditis, cardiac myxoma, atherosclerosis of the aorta, chronic or paroxysmal atrial fibrillation. (12 lead ECG, transesophageal echocardiography, >24-hour ECG monitoring in case of suspected arrhythmias).

Peripheral Vascular System: significant atherosclerosis or dissection of the aorta (TEE, MR, CT).

***Cerebrovascular System: ***clinically relevant atherosclerosis or dissection of the intra- and extracranial arteries (Duplex ultrasound of the carotid arteries, contrast MR or CT head scan). Any preexisting neurological disorder or significant intracranial disease (i.e. multiple sclerosis, arteriovenous malformations, previous intracranial hemorrhage).

***Vasculitis: ***significant collagen vascular disease, giant cell arteritis, vasculitis, systemic necrotizing vasculitis (history, physical examination, erythrocyte sedimentation rate, C-reactive protein, antinuclear antibodies).

***Hematologic: ***hyperviscosity syndromes (erythrocytosis with hematocrit >50%, leucocytosis with white blood cell count >150 000 per μl, thrombocytosis with platelets >106 per μl, paraproteinemia), hypercoagulable states (coagulation status including prothombin time, INR, activated partial thromboplastin time, complete blood cell count, serum protein electrophoresis, anticardiolipin antibodies)

b. Contraindication for chronic oral anticoagulant or antiplatelet therapy:

1. severe bleeding disorder within past 3 months prior to randomization: gastrointestinal bleeding, gross hematuria, known coagulopathy, platelet disorder.

2. significant retinopathy (hemorrhages, exudates)

3. significant intracranial disease

4. previous intracranial hemorrhage

c. Patients who are on chronic anticoagulant therapy for another disease entity (e.g. prosthetic heart valve) other than paradoxical embolism

d. Previous surgical or percutaneous PFO closure

e. Drug or alcohol abuse <48 hours prior to the thromboembolic index event.

f. Septicemia or severe localized infection

g. Pregnancy

h. Severe central nervous system disease (seizure disorder, inflammatory disease of the central nervous system, severe disability from previous stroke, i.e. Barthel-index <50, Modified Rankin scale >3)

i. No informed consent

j. Follow-up over the next 5 years not possible (i.e. severe comorbid diseases with limited life expectancy, unreliable patient etc).

## Appendix 4: Possible adverse events with both treatment modalities

### PFO-device related adverse events

#### Major procedural complications

1. Death occurring within 24 hours of the procedure due to a perioperative complication

2. Cardiac perforation with or without tamponade if requiring pericardiocentesis or surgery

3. Device embolization as dislocation of the PFO device from the atrial septum to another intravascular region necessitating its percutaneous or surgical removal

4. Severe bleeding complication - vascular access site injury requiring surgical correction or significant drop in hemoglobin (>5 g/dl) or requiring transfusion.

5. Intraprocedural stroke (with symptoms persisting for more than 24 hours)

#### Minor procedural complications

1. Cardiac arrhythmias: sustained cardiac arrhythmias related to device implantation requiring electrical (direct current countershock, pacing) or antiarrhythmic drug therapy. Minor arrhythmias (nonsustained, not requiring treatment) are not considered.

2. Cardiac perforation without need for treatment

3. Vascular access site complications

- local hematoma associated with a palpable mass >4 cm and hemoglobin drop >3 and <5 g/dl.

- arteriovenous fistula

- false aneurysm

4. Air embolism with symptoms persisting for >15 minutes but <24 hours

5. Complications necessitating device removal

6. Transient neurologic deficit (TIA) with complete resolution within 24 hours

##### b. Nonprocedural complications

All other adverse events not related to the immediate device implantation period (>24 hours after PFO closure) are considered nonprocedural complications.

- death

- stroke

- peripheral embolism

- need for prolonged hospitalization or readmission

- bleeding complications

### Adverse events related to antithrombotic therapy

- any event leading to interruption of antithrombotic therapy

- bleeding complications related to antithrombotic therapy

## Competing interests

BM has received educational grants from AGA Medical. The other authors have no competing interests.

## Authors' contributions

AAK drafted this manuscript. SW conceived and designed the research. PJ analyzed and interpreted the data. DHS, DD, HRA, RI, GS, ASW, AW acquired the data. HPM conceived and designed the research and made critical revision of the manuscript for important intellectual content. BM conceived and designed the research, made critical revision of the manuscript for important intellectual content and handled funding and supervision. The authors read and approved the final manuscript.
